# Discovery of the Male Acarus Scabiei

**Published:** 1852-02

**Authors:** 


*Discovery of the Male Acarus Scabiei.*—One of M. Cazenave's pupils, M. Lanquetin, has just found the male acarus scabiei upon the hand of a patient affected with the itch. It seems that this acarus had long been sought for in vain, and some works on skin diseases do not even mention its existence. As this parasite is very small, being less than half the size of the female, it had hitherto escaped detection.—*L'Union Médicale.*

